# Evaluating the nutrition and body mass index clinical link pathway in mental health and learning disability services: A mixed-methods study

**DOI:** 10.1371/journal.pone.0303893

**Published:** 2024-06-13

**Authors:** Emma L. Giles, Heidi Stevens, Grant J. McGeechan, Lauren Walker, Narut Pakunwanich, Vicki Whittaker, Jo Smith

**Affiliations:** 1 School of Health and Life Sciences, Teesside University, Middlesborough, United Kingdom; 2 School of Social Sciences, Humanities, and Law, Teesside University, Middlesborough, United Kingdom; 3 Lancaster Medical School, Faculty of Health and Medicine, Lancaster University, Lancaster, United Kingdom; 4 The Research and Development Team, Tees, Esk and Wear Valleys NHS Foundation Trust, Middlesbrough, United Kingdom; University of Maribor, SLOVENIA

## Abstract

This research involved an evaluation of the Nutrition and Body Mass Index Clinical Link Pathway (NBMI CLiP) implemented in practice across Severe Mental Illness and/or learning disabilities ward in Tees, Esk and Wear Valleys NHS Foundation Trust (TEWV), to understand how the NBMI CLiP is used, inpatient staff feedback on the CLiP for supporting service users to manage their weight, and whether using the NBMI CLiP impacted on staffs’ own weight management. To account for the uneven distribution of the secondary data, descriptive statistics such as medians and the inter-quartile range were conducted to assess anychanges in recording of Body Mass Index, nutrition screening (SANSI) and intervention planss. Staff survey data investigated barriers and facilitators to using the NBMI CLiP in practice and the impact on their own weight management. Secondary data analysis found most wards improved recording of BMI, SANSI and Intervention Planning. Forensic Learning Disabilities, Adult Learning Disabilities, mixed gender wards and North Yorkshire and York Operational Directorate indicated the greatest improvement. Survey results (n = 55) found three times as many participants (n = 12, 75%) found the NBMI CLiP easy or very easy to use; most fully understood it (n = 13, 81.20%) and were confident or very confident to carry out a SANSI Screen (n = 14, 87.50%) or a recovery focused intervention plan (n = 9, 56.20%). Open-text responses, analysed using content analysis, indicated a need for further training of staff on the NBMI CLiP. It is recommended that to support weight management across these wards, that a nudge or choice architecture approach to weight management is adopted, supported by training delivered by a dietitian.

## Introduction

Overweight and obesity are non-communicable diseases associated with a range of co-morbidities which can impact on health-related quality of life. Worldwide over one billion people will be overweight or have obesity by 2025 [1]. In England, it is currently estimated that between 59–66% of the adult population have overweight or obesity [2]. Individuals with mental ill-health are a marginalised population in terms of physical health conditions, many of which are preventable. People with Severe Mental Illness (SMI), which includes schizophrenia, bipolar disorder and other psychoses, are particularly vulnerable in terms of their physical health, which may result in a shorter life expectancy of 15–20 years compared to the general population [3]. Additionally, people with learning disabilities (LD), including Downs’ syndrome, Fragile X syndrome, and William’s syndrome [4, 5], have a shorter life expectancy of between 23 to 27 years when compared to the general population [4, 6]. In England, adults with SMI are almost twice as likely to be living with obesity than the general population [7], while it is estimated that 37% of people with LD are classified as having obesity compared to 30% of the general population [8]. For individuals with SMI and/or LD, it can be challenging to manage their weight. This can be due to a range of modifiable risk factors, for example inadequate physical activity, particularly for in-patients on mental health units [6, 9]; second generation antipsychotics which can cause hyperphagia, metabolic dysregulation and rapid weight gain [10]; poor diet due to, for example, a lack of access to healthy food [4, 8, 11, 12]; consumption of unhealthy take-aways or microwaveable meals [13, 14]; the use of food as a maladaptive coping mechanism due to low mood or depression [11, 15, 16]; or a lack of knowledge about healthy living [8, 11]. Recent research has also shown that people with SMI have a higher intake of sweetened beverages [17], cakes, sweets, hydrogenated oil and fast food, and consume- fewer fruits and vegetables than people without SMI [18].

To help support weight management in people with SMI and/or LD, a regional weight management plan for people with SMI and/or LD was developed called ‘A Weight off Your Mind’ (AWoYM) [19]. AWoYM was launched in 2018 across the two main mental health Trusts in the North of England: Tees, Esk, and Wear Valleys NHS Foundation Trust (TEWV) and Cumbria, Northumberland, Tyne and Wear NHS Foundation Trust (CNTW). This was a joint initiative between Public Health England, NHS England’s Clinical Networks and the two NHS Trusts. Through co-production with experts-by-experience (with lived experience of SMI), ten components of AWoYM were developed, and included elements on: leadership, physical health screening, food and nutrition, improved access and opportunity for physical activity, pharmacy/medicines management, psychological therapies, education and information, developing community pathways, children and young people, and LD. This paper reports an evaluation of one component of the AWoYM plan, that of the Nutrition and Body Mass Index Clinical Link Pathway (NBMI CLiP) that was implemented in practice in TEWV. At the time of the evaluation, the NBMI CLiP was one component of the AWoYM plan which was used across 47 wards in TEWV to help inpatients to manage their weight (patients with, or at risk of developing a BMI<20Kg/m^2^ or ≥25Kg/m^2^). Inpatient wards provided care for patients with a range of mental health diagnoses, including common mental illness, SMI, LD, autism or dementia. Furthermore, some patients were detained in a secure (forensic) unit under the Mental Health Act (1983) [20].

The NBMI CLiP involves a nutrition screen using the St Andrew’s Healthcare Nutrition Screening Instrument (SANSI). Service users who are identified as medium or high risk are guided by healthcare staff to develop a person-centred weight management intervention plan through a range of evidence-based interventions that stem from the AWoYM plan. The intervention categories in the NBMI CLiP are: 1. Information 2. Dietary Change and Education 3. Physical Activity and Exercise 4. Psychological Interventions 5. Signposting and 6. Pharmacology. Service users chose a combination of intervention categories that they feel will be most effective for them as an individual. This is the stark difference between this intervention and previous published weight management interventions in people with mental illness and/or learning disabilities, with limited research on how the NBMI CLiP can support person-centred care and its perceived impact by staff.

The aim of this research was to undertake a process evaluation of the NBMI CLiP. The specific objectives were to understand how the NBMI CLiP is used to understand inpatient staff feedback on the CLiP for supporting service users with mental illness (including SMI) and/or LD to manage their weight; and whether using the NBMI CLiP in practice has any impact on staffs’ own weight management. Specifically, this paper reports on findings related to implementation of the NBMI CLiP, staff feedback, and wider suggestions for how to support inpatients and staff to manage their weight.

## Methods

### Ethical approval

This study was conducted according to the guidelines laid down in the Declaration of Helsinki and all procedures involving human subjects/patients were approved by the School of Health and Life Sciences Research Ethics and Governance Committee at Teesside University and the Health Research Authority (HRA) for the secondary data analysis (IRAS project ID: 292827) and for both the pilot and main surveys (IRAS project ID: 285589). Furthermore, Research and Development approval was obtained from TEWV following HRA approval, and the appropriate letters of access were issued to research staff. Consent was implied by submission of the survey.

### Secondary data analysis

Data on routinely collected anonymised Key Performance Indicators (KPIs) were securely provided by TEWV as an anonymous Excel spreadsheet. Access to the data were facilitated by TEWV staff. At no point was patient or ward-level data accessed by the research team, as data had been assigned IDs prior to transfer. Data on the following KPIs were obtained from the electronic patient record (PARIS), and reported on a monthly basis for each ward: 1) Percentage (%) of patients with a BMI recorded, 2) Percentage (%) of patients with a St Andrew’s Nutrition Screening Instrument (SANSI) recorded, and 3) Percentage (%) of patients with an intervention plan recorded (for service users identified as being medium or high risk on the SANSI).

The data covered the period from the implementation of the NBMI CLiP in October 2018 until March 2021 and was accessed on the 21^st^ April 2021. Baseline data represented the month before implementation of the NBMI CLiP for each ward. Some wards implemented the pathway later than others, therefore baseline data represented different dates. Therefore, wards had varying numbers of time-points (months) for analysis. As such, the months following baseline are represented as time-points 1 to 30 (T1 to T30). There were also some time-points where data had not been recorded due to staff shortages.

The data were imported from the spreadsheet and analysed using SPSS v26. Prior to analysis data were cleaned; removing closed or new wards without baseline data. Data from 47 wards were included in the analysis (completed 4^th^ October 2021). To determine specific factors associated the with recording of outcomes, data were analysed in the following sequence for all three KPIs:

1. Individual ward-level analysis (n = 47);
2. Aggregate analysis for all wards (n = 47) to assess overall Trust performance; KPI.
3. Subgroup analysis by ward speciality: Adult Mental Health (AMH), Adult Learning Disabilities (ALD), Mental Services for Older People (MHSOP), Forensic Learning Disabilities (FLD) and Forensic Mental Health (FMH); 4. Subgroup analysis by sex (men, women, mixed);
5. Subgroup analysis by geographical directorate (Teesside, Durham & Darlington, North Yorkshire, York & Selby, and Forensics).

Summary and descriptive statistics were calculated for the three KPI separately. Histograms and associated tests of normality were conducted to determine the viability of performing parametric statistical testing. The Kolmogorov-Smirnov test of normality indicated violation of the assumption of normality (p<0.05) for the data set therefore, paired t-tests and repeated measures ANOVA were not conducted. Additionally, due to different implementation dates which introduced large imbalances in the data across wards it was decided that statistical testing would be inappropriate. To account for the uneven distribution in the data, medians the Inter-Quartile Range IQR) were calculated instead of means and standard deviation. Minimum and maximum values were also included to indicate which wards were performing well and which needed attention. Medians (IQR) for recording of BMI, SANSI and intervention plan were graphically displayed using tables and box plots.

### Survey

The survey was developed to gather quantitative and qualitative data regarding participants’ views on the facilitators and barriers to using the NBMI CLiP, and to explore whether their use of the NBMI CLiP impacted on their personal weight management. The survey included a quasi-qualitative approach of multiple-choice questions, analysed using descriptive statistics and open ended free-text questions, analysed using content analysis, which gave participants the opportunity to expand on closed questions [21]. A pilot of the main survey was undertaken between July and August 2020 (by NP) on two wards (n = 15 participants). Participants in the pilot were asked a short series of questions at the end of the survey to rate the length and question acceptability. Eighty-seven percent of participants stated the survey was the right length and 93% indicated they understood the questions. No changes were made to the survey prior to data collection, and the pilot data were therefore included in the analysis [21].

Following the pilot survey (22^nd^ July 2020 to 14^th^ September 2020), the survey was circulated online across between 14^th^ March and 30^th^ June 2021 to all staff working in 47 inpatient wards, including nursing, medical, and assistant staff and Allied Health Professionals. Participants were inpatient staff aged 18 years and over. Recruitment of inpatient staff was challenging due to the significant pressures on inpatient services, as a result of COVID-19 and multiple Care Quality Commission inspections that were taking place on inpatient units. Therefore, a range of methods were used to increase participation in the survey, with ethical approval obtained for any alterations to the protocol. Potential participants were identified through the Modern Matrons and later through the Electronic Staff Record and approached by email to participate in the online survey. The study was also promoted on the Trust weekly e-bulletin email and the staff intranet site. The sample size aimed to recruit 50 participants from the 2,590 identified staff members. As the research formed part of an evaluation, a formal sample size calculation was not required.

The survey platform used was Jisc Online Surveys to which Teesside University is subscribed. This avoided face-to-face contact, both to protect anonymity and promote social distancing due to COVID-19. Participant consent was implied via completion of the survey. The Participant Information Sheet (PIS) was included as a link at the top of the survey, providing full details of the study and researcher contact details.

## Results

### Secondary data analysis

A summary of the wards included in the analysis can be found in Table 1 to provide clarity on which wards were performing well and which needed improvement.

**Table 1:** Ward summary.

Ward specialty	Wards n, (%)
Adult Mental Health	17 (36.17)
Adult Learning Disabilities	3 (6.38)
Forensic Mental Health	12 (25.53)
Forensic Learning Disabilities	7 (14.89)
Mental Health Services for Older People	8 (17.02)
**Total**	**47 (100)**
**TEWV Operational Directorate**	
County Durham and Darlington	13 (27.65)
Forensic Services	19 (40.42)
North Yorkshire and York	6 (12.76)
Teesside	9 (19.14)
**Total**	**47 (100)**
**Gender of the inpatients on each ward**	
Women only	12 (25.50)
Men only	20 (42.60)
Mixed	15 (31.90)
**Total**	**47 (100)**

*To determine the change in the recording of BMI compared to pre-intervention data*, found that 14 wards demonstrated 100% recording of inpatients’ BMI both at baseline and at T30. Nine wards showed an increase in recording of BMI between baseline and T30, seven of which increased to 100%, but there was a decrease in recording of inpatients’ BMI across nineteen wards between T1 and T30. The ward with the lowest percentage recording overall at T30, decreased from 100% at baseline to 25% at T30. Results for the recording of inpatients’ BMI stratified by ward specialty found that across ward specialties, the ALD wards maintained a consistently high median 100% recording of BMI across all time-points. Across the FM wards, median recording of BMI was high both at baseline (100%, IQR: 89,100) and at T30 (100%, IQR: 74,100). Similarly, across the FLD wards, median recording of BMI was also high both at baseline (100%, IQR: 100,100) and at T30 (100%, IQR: 67,100). In contrast, across the AMH wards, there was a decrease in median recording of BMI between baseline (94%, IQR: 89,100) and T30 (82%, IQR: 69,100). Additionally, across the MHSOP wards there was also a decrease in median recording of BMI between baseline (97%, IQR: 89,100) and T30 (90%, IQR: 83,100).

*To assess the change in the recording of nutrition screening (St Andrew’s Healthcare Nutrition Screening Tool or SANSI) of service users across TEWV inpatient wards*, there was improvement in the recording of inpatients’ SANSI across most wards in TEWV between baseline and T30 (n = 40); with 18 of these wards increasing from 0% at baseline to 100% at T30. Only two wards (AMH and MHSOP) demonstrated a decrease in the recording of SANSI between baseline and T30. The wards across ALD showed the greatest improvement from baseline (0%, IQR: 0,0) to T30 (100, IQR: 100,100). Across the AMH wards there was also overall improvement from 0% (IQR: 0,0) at baseline to 89% (IQR: 81,99) at T30. Across the FMH wards there was improvement from 0% (IQR: 0,0) at baseline to 100% (IQR: 75,100) at T30, and across the FLD wards there was improvement from baseline (0%, IQR: 0,0) to T30 (100%, 28 IQR: 82,100). Similarly, across the MHSOP wards there was also improvement from baseline (38%, IQR: 0, 88) to T30 (87%, IQR: 74,100).

*To assess the changes in intervention planning for service users who are identified as being medium or high risk on the Nutrition and BMI Clinical Link Pathway (NBMI CLiP)*, seven wards maintained 100% recording of inpatients’ Intervention Plans (IPs) at baseline and at T30. Nineteen wards increased recording of IP between baseline and T30, four of which increased to 100% at T30. Sixteen wards decreased recording of IP between baseline and T30, ranging from 13% to 91% at T30. The recording of inpatients’ IP by TEWV Operational Directorate showed that ALD wards improved from baseline (100%, IQR: 75,100) to T30 (100%, IQR: 100, 100) as did MHSOP with improved recording of IP from baseline (39%, IQR: 27,69) to T30 (50%, IQR: 41,64). At T30 within MHSOP there was one outlying ward with a low recording of IP; however, AMH showed the greatest improvement from baseline (12%, IQR: 0,47) to T30 (63%, IQR: 49,69), and at T30 there was one only ward in AMH with a low recording of IP. FLD wards maintained a high median recording of IP at baseline (100%, IQR: 88,100) and at T30 (100%, IQR: 57,100). Only FMH wards showed a slight downward trend from baseline (94%, IQR: 77,100) to T30 (83%, IQR: 72,100).

### Survey

The survey of NHS TEWV staff was developed to address the following two research objectives:*To gather quantitative feedback from staff on their views regarding the facilitators and barriers to using the NBMI CLiP to support service users in managing their weight*, and *To explore with staff whether their use of the NBMI CliP has motivated them to manage their own weight*. Table 2 shows the participant characteristics of those who completed a survey (n = 55).

**Table 2:** Survey participant characteristics.

Q1 Age, mean 42.80 years (SD: 12.80; min, max 21–75)	
**Q2 Gender**	**n (%)**
Man	10 (18.20)
Woman	45 (81.80)
Other	0 (0)
Prefer not to state	0 (0)
**Q3 Role**	
Modern Matron	2 (3.60)
Clinical Lead	3 (5.50)
Staff Nurse	12 (21.80)
Associate Practitioner	4 (7.30)
Health Care Assistant	22 (40.0)
Allied Health Professional	5 (9.10)
Doctor	2 (3.60)
Apprentice nurse	1 (1.80)
Administrator	1 (1.80)
Assistant psychologist	1 (1.80)
Associate nurse consultant	1 (1.80)
Ward clerk	1 (1.80)
**Q4 Ward**	
Adult Mental Health	17 (30.90)
Adult Learning Disabilities	13 (23.60)
Forensic Mental Health	9 (16.40)
Forensic Learning Disabilities	5 (9.10)
Mental Health Services for Older People	11 (20.00)
**Q5 Employment experience in a mental health/LD unit**	
0–1 year	9 (16.40)
2–5 years	13 (23.60)
6–10 years	11 (20.00)
11–15 years	5 (9.10)
16 + years	17 (30.00)
**Q6 Involvement in the NBMI CLiP**	
Regularly	14 (25.50)
Occasionally	2 (3.60)
Aware but not directly used	9 (16.40)
Unaware	30 (54.40)

Three times as many participants (n = 12, 75%) (Table 3) found the NBMI CLiP easy or very easy to use than those who selected neutral or found it hard to use (n = 4, 25%). The majority fully understood it (n = 13, 81.20%) and were confident or very confident to carry out a SANSI Screen (n = 14, 87.50%) or a recovery focused intervention plan (n = 9, 56.20%).

**Table 3:** Ease of use of the NBMI CLiP.

**Q7 How easy do you find the NBMI CLiP to use** **n (%)**	**Very easy**	**Easy**	**Neutral**	**Hard**	**Very hard**
	6 (37.50)	6 (37.50)	3 (18.75)	0 (0)	1 (6.25)
**Q8 Do you agree that you fully understand the NBMI CLiP?** **n (%)**	**Strongly agree**	**Agree**	**Neutral**	**Disagree**	**Strongly disagree**
	3 (18.75)	10 (62.50)	1 (6.25)	1 (6.25)	1 (6.25)
**Q9 How would you rate your confidence in carrying out a SANSI Nutritional Screen?** **n (%)**	**Very confident**	**Confident**	**Neutral**	**Unconfident**	**Very unconfident**
	9 (56.25)	5 (31.25)	1 (6.25)	0 (0)	1 (6.25)
**Q10 How would you rate your confidence in producing recovery focused intervention plans to support service users? n (%)**	**Very confident**	**Confident**	**Neutral**	**Unconfident**	**Very unconfident**
	4 (25.00)	5 (31.25)	5 (31.25)	1 (6.25)	1 (6.25)

When the responses to the use of the NBMI CLiP were stratified by role, results indicated that all Allied Health Professionals and Associate Practitioners (31.25%) found the NBMI CLiP very easy or easy to use, while only one (6.25%) participant who was a Staff Nurse found the NBMI CLiP very hard to use. When stratified by ward speciality only one participant (6.25%) who worked in FMH found the NBMI CLiP hard to use, all other participants chose the neutral to very easy options (81.25%). Similarly, when stratified by years of service only one participant (56.25%) with 6–10 years’ service found it hard to use. The SANSI was perceived to be the most useful element of the NBMI CLiP by 11 (68.75%) participants (Table 4).

**Table 4:** Most and least useful elements of the NBMI CLiP.

Participants (n)of the NBMI CLiP	Most useful n(%)	Least useful n(%)
None of it	3 (18.75)	n/a
All of it	n/a	2 (12.50)
The SANSI	11 (68.75)	1 (6.25)
The Resources to Support Intervention Planning Where Required	5 (31.25)	0 (0)
The Dietary Change and Education Resources	5 (31.25)	0 (0)
The Physical Activity and Exercise Resources	3 (18.75)	3 (18.75)
The Information for Service Users	2 (12.50)	2 (12.50)
The Psychological Interventions and Therapies Resources	2 (12.50)	0 (0)
The Signposting Resources	2 (12.50)	3 (18.75)
The Review Section	2 (12.50)	3 (18.75)
The Pharmacology Resources	1 (6.25)	3 (18.75)
Q11 other (free text): ‘Support from the dietitian one of the most proactive dietitians I’ve met’.	1 (6.25)	n/a
Q12 other (free text): ‘Absolutely none of it’.	n/a	1 (6.25)
Q12 other (free text): ‘The tool itself is based on an outdated model’.	n/a	1 (6.25)

Half of the participants answered that using the NBMI CLiP in practice had made them consider their own weight, while three participants (18.75%) thought they had no issues with their weight management. One participant who had considered their own weight management added that they thought the NBMI CLiP was a ‘great tool’ and would like to use it for their own weight management while two participants had already made active changes to be healthier. Two participants left free text answers, one of which stated that the NBMI CLiP did not take into consideration body types, body composition or hidden disabilities that made exercising and losing weight challenging. One of these participants showed concern with regard to the potential for subsequent eating disorders and mental health issues. Six participants thought that they both understood the NBMI CLiP and that using it in practice had made them think about their own BMI, whilst two participants thought that they understood the NBMI CLiP enough to actively made changes to be healthier. Only one participant was not interested in their BMI and this participant had strongly disagreed that they understood the NBMI CLiP.

Four participants (25%) used resources from the NBMI CLiP for their own weight management, referring to the physical activity and exercise resources, SANSI, mindful eating information and psychological therapies. The SANSI was chosen as the most useful of the resources to implement in practice. Additionally, most participants (n = 45, 81.8%) were unaware that TEWV offered weight management groups for staff, with only one participant attending a weight management group and this participant reported that they had lost weight by the end of the sessions. Nine participants were aware of TEWV weight management groups but did not attend, and of these, five also used the NBMI CLiP in practice which does not indicate any association between using the NBMI CLiP and awareness of TEWV staff weight management groups.

Furthermore, of the 55 participants, 51 (92.73%) left free-text responses in the survey. There were many comments expressing a need for more training on the NBMI CliP and five participants thought that training on the NBMI CLiP should be essential for all new staff including new HCAs, *‘training should be part of induction’*. Most participants who commented on the need for more training on the NBMI CLiP commented that this would have been helpful before they had started using the Pathway, *‘I used the CLiP for a long time before I had any training’*. However, some participants also stated that training would still be beneficial and made specific suggestions such as dietitian involvement, case studies with demonstrations and *‘training in this and on how to record correctly on PARIS’* (the Trust’s computer system).

Two participants made comments relating to accessibility where information had to be adapted for older service users living with ‘*cognitive issues’* while another participant stated that any information about the NBMI CLiP should be in easy read. Other participants also referred to a lack of patient interest in weight management on the part of some service users refusing to be weighed. One participant thought it should be *‘promoted as exciting’* or adding *‘a bit of fun to a project attracts more interest’*.

The majority of participants made comments about their own weight management (n = 38, 69.09%). Comments pertained to a lack of awareness regarding existence of the weight management groups but there were also expressions of interest in attending one, *‘I didn’t know it existed I’d love to’*. There were also proactive suggestions on how to publicise weight management groups for staff, such as posters/visual displays in restrooms, online links, information at inductions and circulation of information packs at staff meetings.

Comments were made relating to convenience in terms of the proposed weight management groups fitting into the workday, being online or held locally, *‘Making them very local and frequent*.*—Including time into workday’*. Staff were concerned about having no time for weight management due to the busy nature of the workplace, *‘Time off the ward*, *which we often cannot get due to it being a fast-paced busy ward’*. Participants also offered a variety of suggestions such as ‘*staff incentives or work-based support’* to facilitate attendance at weight management groups or *‘encouragement from bosses to attend’*. Incentives to facilitate physical activity included access to gyms with one participant mentioning that this was already offered to staff across some TEWV sites, *‘[site] has a gym staff can access after patient use other TEWV sites should have one I honestly think it’d be popular and it’ll great for mental health wellbeing’*. A further suggestion was partnering with local authorities for discounted use of fitness facilities, ‘*Partner with local authorities for cheaper fitness classes swimming etc’*. One participant suggested offering *‘counselling regarding reasons for weight gain*, [and] *poor body image*, *emotional eating’*, which all align with the holistic approach of AWoYM. Other practical ideas included ‘*weight monitoring’* with ‘*dietetic input’*, providing *‘shower areas for staff who want to use the gym’*, sponsoring staff to take part in charitable events, sports events and team lunches. It was also stated that colleagues could offer one another support to attend weight management groups.

Finally, the work environment was perceived as a barrier to leading a healthy lifestyle by some particularly in terms of diet, where it was thought that staff were not given sufficient time to eat during the workday and healthy food options were not available in the canteens or on the units, ‘*healthy canteen food or a canteen serving healthy food would be good so we don’t have to bring ’fast food; to work or order takeaways in a 12 hour shift’*. Other food related suggestions included discounts on healthy food and ‘*life without takeaways’*. One participant also stated that there was no food provision for a vegan diet within the hospital and another suggested ‘*TEWV funded lunches should only contain whole foods*, *predominantly plant based’*.

## Discussion

The aim of this research was to conduct a mixed methods process evaluation of the TEWV AWoYM intervention to understand the impact of the NBMI CLiP on the weight management of service users with mental illness and/or LD, and staff perceptions on how best to support service users, and themselves, to manage their weight effectively.

Recording of BMI across TEWV showed only a very slight decrease but was still high at the last time-point (median 97%). This aligns with the Adult Secure Clinical Reference Group and Managing a Healthy Weight Task group recommendations that physical health information such as BMI is recorded on admission and on a regular basis [22]. This is also consistent with other findings; an audit of physical health monitoring in adult psychiatric inpatient wards across an NHS Foundation Trust, found 91% of patients had their BMI recorded within twenty-four hours of admission [23]. Similarly, data from the Quality and Outcome Framework (QOF) showed a rate of 84% for recording of BMI for patients with SMI across general practices in the UK [24]. In contrast, other data has shown recording of BMI across mental health providers to be inconsistent and varied ranging from 5% to 92% [25], this suggests that recording of BMI has been implemented into practice across TEWV successfully.

There were positive results for use of the SANSI in practice where recording of SANSI showed the greatest improvement across wards from baseline to the final time-point. This was also consistent with results from the survey that indicated most staff who used the NBMI CLiP in practice were confident/very confident in carrying out a SANSI screen. Additionally, the SANSI was also perceived by staff as the most useful element of the NBMI CLiP. This suggests that the SANSI has been implemented into practice across TEWV successfully, which aligns with the Adult Secure Clinical Reference Group and Managing a Healthy Weight Task group recommendations that staff working in an adult secure mental health setting should be skilled in using a relevant nutritional screening tool [22].

A similar result was found in a pilot study of the SANSI in a psychiatric setting, with findings indicating that not only was it a reliable, consistent and valid screening tool but it typically took less than ten minutes for ward staff to use and resulted in the screening of 93% of service users [26]. Overall, the SANSI was found to be both an appropriate and acceptable element of the NBMI CLiP, which is crucial as the SANSI is the first line approach to assessing the nutritional status of service users to determine whether they are at risk of overweight or obesity.

Overall, there was improvement in recording of intervention plans from baseline to the last time-point (median 70%) which indicates that the IP is being implemented well. Indeed, it is recommended that all adult secure mental health providers in the UK should have a treatment program for service users with obesity [22], however, health screening and monitoring has been reported as inadequate and inconsistent in practice [25]. Recommendations further stipulate that interventions should encompass essential elements such as diet, nutrition, physical activity, structured exercise, medication, treatment and behavioural science [22] and holistic and personalised approaches that allow the service user to prioritise areas they need to work on [22], which the IP element of the NBMI CLiP aims to achieve. Service users in secure hospitals experience many issues that make it necessary to adapt guidelines specifically to suit this population. As opposed to a one size fits all approach, the multi-component IP is tailored to service users with SMI and/or LD and empowers service users to coproduce their own IP.

Dietitian involvement either in training or in delivery of the NBMI CliP was perceived as positive in survey results. Similar to the findings of our evaluation, a systematic review found dietitian involvement in nutrition interventions and individualised assessments for people with SMI was associated with a larger effect than interventions led by other health professionals for reductions of weight and BMI [27]. It may be worth conducting a future evaluation of how dietitian involvement in delivery of AwoYM across TEWV is perceived from a service user perspective in order to better understand how to support patients.

Within the dietary change and education guidance section of the NBMI CLiP [28] staff are advised to discuss healthy options and healthy snacks on the wards to promote weight loss with service users. Similarly, the Care Quality Commission (CQC) recommends offering advice and encouragement for following a healthy well-balanced diet [29]. In contrast to current recommendations, free text comments made in the survey pertained to a lack of healthy food provision across TEWV and the abundance of unhealthy options readily available to inpatients at hot drinks stations and in vending machines. Guiding service users towards an improved nutritional intake may be challenging to promote where only unhealthy food products are provided, although this issue is challenging to tackle as according to CQC guidance, restricting access to specific food products could be perceived as a blanket restriction which limits personal liberty and rights [30]. Similar findings from a cross-sectional study based in an NHS paediatric hospital indicated only 10% of food options available in vending machines were classified as healthy [31]. A further study of parental perceptions of food choices within a children’s hospital in the North-East of England also found a lack of affordable healthy options on sale although participants did not support an outright ban on unhealthy options either [31].

One possible solution was investigated in a pilot intervention that was conducted within an NHS hospital-based setting and which implemented a choice architecture approach to nudge people into choosing healthier options from hospital vending machines [32]. Such an approach could potentially be piloted across TEWV rather than placing restrictions on specific food products. This could be important not just from a weight management perspective but also to comply with mandatory hospital food standards in the NHS that stipulates all hospitals should develop a food and drink strategy which includes healthier eating for the whole hospital community including staff [33].

Additionally, within the survey some participants expressed comments that there was a lack of accessible materials to cater for differences in the capacity of service users with LD or older patients with cognitive issues. The AWoYM plan does stipulate that all materials should be easy to understand and available in an easy-to-read format. Some materials such as the ‘My Goal Setting Pack’ which is an element of Intervention Planning are presented in an easy read format, however others such as BDA Food Fact Sheets to which service users are signposted are only available in a standard format [34]. This suggests that some of the NBMI CLiP materials may require reassessing and updating ideally with service user coproduction. Research shows coproduction with relevant individuals and communities may highlight barriers, facilitators and solutions that may not otherwise be identified by researchers [35]. A reassessment of materials could align AWoYM with the mandatory Accessible Information Standard which aims to make sure that people who have a disability or sensory loss are given information by the NHS and adult social care in a way they understand [36]. Different formats of information such as easy read, British Sign Language and audio are readily available on the AWoYM website for community access, but this is not mirrored consistently for inpatients across the NBMI CLiP [28].

Furthermore, half of the participants who responded in the survey thought that using the NBMI CLiP in practice had made them think about their own weight, but a large majority were unaware that TEWV offered staff weight management groups. There was a desire to participate in staff weight management programmes amongst participants but also a consensus that not enough information was circulated about the available programmes. It was suggested by participants that staff weight management groups should be better advertised and that incentives could be offered to help staff manage their weight such as more peer support. Participants also found eating healthy challenging due to time constraints during the workday, although the NHS Health and Wellbeing Strategic Overview [37] emphasises the need for proper spaces for staff to rest and take breaks. The NHS Health and Wellbeing Strategic Overview [37] places staff health and wellbeing at the top of its agenda with emphasis on targeted preventative interventions and maintaining life balance. In line with this there are clear weight management interventions available for staff across TEWV including online options, but these may need clearer and wider promotion.

Some participants in the survey thought that more training was needed on the NBMI CLiP, especially for new members of staff and HCAs. According to the NBMI CLiP Required Skills Matrix [38] training on the SANSI and IP is essential for staff working in certain roles such as Dietitians or Nurses, in contrast for HCAs training is classed as desirable but not high priority. This perhaps warrants further investigation to assess whether HCAs should be included consistently in training on the NBMI CLiP. There has been a rising number of HCAs working within the UK during the past twenty years allowing Nurses to focus on patient care [19]. Some HCAs in our results also described having no knowledge of how to perform certain tasks and learning through experience [39] which again perhaps demonstrates a gap for standardised and structured training for HCAs across the NHS.

Lastly, it was commented by some participants within the survey that provision for dietary requirements was lacking. Religious dietary requirements including the vegan diet are protected characteristics. Furthermore, ‘The Equality Duty in Practice’ requires all public sector services to meet different people’s needs [40]. Additionally, according to the British Dietetic Association (BDA) KPI/Department of Health Hospital Food Standards, cultural and dietary food choices should be made available to hospital patients where required [41]. Being admitted to hospital may be a stressful experience for some people while a lack of provision for dietary requirements can be an additional stressor [42]. It is therefore important that hospitals meet religious and cultural requirements and that staff have the appropriate knowledge [43]. Of the TEWV service user population 2.39% are from an ethnic minority background [44], therefore it may be worthwhile involving this patient group in research to determine what cultural and dietary requirements need to be catered for if any. This would align with the TEWV Equality, Diversity and Human Rights Strategy 2020–2023 [44] which aims to improve equality and diversity for service users, carers and staff. It was also discussed by some interview participants that staff lacked knowledge about cultural and dietary requirements; cultural knowledge is the process of seeking and obtaining sound educational foundation about diverse cultural and ethnic groups [45]. This suggests that although cultural knowledge is improving, further policies need to be implemented that support staff awareness and provision of cultural and religious dietary needs.

Overall, this research developed a strong understanding of the use and recording statistics of the SANSI and NBMI CLiP within a mental health setting in the North East of England; important to consider as their AWoYM plan is currently being refreshed. Whilst we sought to obtain a sample that was representative of a wide range of professionals involved in care of patients with SMI, and with good representation across wards, the main limitation of this research includes a limited sample size which limits the scope of findings beyond the local context.

## Conclusion

A mixed methods process evaluation of the TEWV AWoYM intervention involved a survey of NHS TEWV staff views of the facilitators and barriers to using the NBMI CLiP in clinical practice, and data on routinely collected anonymised KPIs, BMI, SANSI, and IP.

Results showed a high level of BMI recording across inpatient units which aligned with official recommendations, indicating successful implementation into practice of BMI recording. SANSI recording showed the greatest improvement across wards and high levels of acceptability from staff regarding its use. This further aligns with official recommendations that staff working in an adult secure mental health setting should be skilled in using a relevant nutritional screening tool. Results also indicated that IP were being implemented well and that its holistic, personalised approach aligns with the need to adapt and co-produce guidelines to inpatients with LD and/or SMI. It was found that dietitian involvement in training or delivery of the NBMI CLiP was perceived as desirable and may warrant a future evaluation of how dietitian involvement in delivery of AWoYM across TEWV is perceived from a service user perspective. A lack of healthy food provision and consideration of dietary requirements was highlighted which ran contrary to guidelines stipulated by both the CQC and the AWoYM plan. Rather than imposing blanket bans on unhealthy food, it is recommended that to address this issue, a nudge or choice architecture approach to food provision be considered. A lack of accessible materials also ran contrary to the AWoYM plan, indicating materials may require reassessing and updating, ideally with service user co-production. Finally, using the NBMI CLiP had prompted many staff to consider their own weight and suggestions were made regarding work-based incentives and improved advertisement of staff weight management groups.
